# Intrinsic Abnormalities of Cystic Fibrosis Airway Connective Tissue Revealed by an In Vitro 3D Stromal Model

**DOI:** 10.3390/cells9061371

**Published:** 2020-06-01

**Authors:** Claudia Mazio, Laura S. Scognamiglio, Rossella De Cegli, Luis J. V. Galietta, Diego Di Bernardo, Costantino Casale, Francesco Urciuolo, Giorgia Imparato, Paolo A. Netti

**Affiliations:** 1 Istituto Italiano di Tecnologia (IIT), Largo Barsanti e Matteucci 53, 80125 Napoli, Italy; claudia.mazio@iit.it (C.M.); laura.scognamiglio@iit.it (L.S.S.); 2 Telethon Institute of Genetics and Medicine (TIGEM), Via Campi Flegrei 34, 80078 Pozzuoli (NA), Italy; decegli@tigem.it (R.D.C.); l.galietta@tigem.it (L.J.V.G.); diego.dibernardo@unina.it (D.D.B.); 3 Department of Chemical, Materials and Industrial Production Engineering (DICMAPI) University of Naples Federico II, P. le Tecchio 80, 80125 Napoli, Italy; costantino.casale@unina.it (C.C.); urciuolo@unina.it (F.U.); 4 Interdisciplinary Research Centre on Biomaterials (CRIB), University of Napoli Federico II, P. le Tecchio 80, 80125 Napoli, Italy

**Keywords:** cystic fibrosis, connective airway tissue, lung fibroblasts, extracellular matrix, 3D bioengineered model, RNA sequencing

## Abstract

Cystic fibrosis is characterized by lung dysfunction involving mucus hypersecretion, bacterial infections, and inflammatory response. Inflammation triggers pro-fibrotic signals that compromise lung structure and function. At present, several in vitro cystic fibrosis models have been developed to study epithelial dysfunction but none of these focuses on stromal alterations. Here we show a new cystic fibrosis 3D stromal lung model made up of primary fibroblasts embedded in their own extracellular matrix and investigate its morphological and transcriptomic features. Cystic fibrosis fibroblasts showed a high proliferation rate and produced an abundant and chaotic matrix with increased protein content and elastic modulus. More interesting, they had enhanced pro-fibrotic markers and genes involved in epithelial function and inflammatory response. In conclusion, our study reveals that cystic fibrosis fibroblasts maintain in vitro an activated pro-fibrotic state. This abnormality may play in vivo a role in the modulation of epithelial and inflammatory cell behavior and lung remodeling. We argue that the proposed bioengineered model may provide new insights on epithelial/stromal/inflammatory cells crosstalk in cystic fibrosis, paving the way for novel therapeutic strategies.

## 1. Introduction

Cystic fibrosis (CF) is a genetic disease caused by mutations in the cystic fibrosis transmembrane conductance regulator (CFTR) gene. CFTR is a chloride channel located at the apical membrane of epithelial cells of different organs where it plays an important role in transepithelial electrolyte and fluid transport [1]. CF is a multi-organ pathology, but lung disease causes most of the severe complications in CF. As result of CFTR dysfunction, CF patients display a chronic respiratory disease with mucus plugging and bacterial colonization of the airways [2]. Persistent cycles of infection activate an intense inflammatory response followed by tissue remodeling and lung fibrosis with increase in fibroblast proliferation and accumulation of extracellular matrix (ECM) elements [3,4]. Despite lung fibrosis being deeply investigated in other pulmonary disease such as asthma, chronic obstructive pulmonary disease, and idiopathic pulmonary fibrosis, it has not well characterized in patients with CF [5]. Indeed, most of the studies about CF are focused on epithelial cells because they express CFTR and are directly compromised by channel dysfunction. For this reason, most of the in vitro models of CF are composed of epithelial cells. At present, several primary epithelial cell models have been developed both in 2D and 3D culture conditions with the aim to model CF pathogenesis and to test drugs for mutant CFTR rescue [6]. Usually, airways epithelia are studied in vitro by seeding bronchial or nasal epithelial cells on porous membranes and inducing their differentiation under air–liquid interface (ALI) conditions. Another class of CF model is represented by the intestinal or pulmonary organoids. They represent hollow epithelial structures developed in 3D matrices such as Matrigel. The organoids allow to easily investigate CFTR function as a simple change in volume. Indeed, stimulation with cAMP agonist such as forskolin induces CFTR-mediated luminal secretion of electrolytes and hence water leading to organoid swelling [7]. The aforementioned models are very successful to study CFTR dysfunction and patient-specific response to drugs aiming to recovery CTFR function [6]. Nevertheless, these models do not consider the impact that stromal components have in CF. Moreover, although CF is primarily an epithelial pathology, the stroma may affect the progression of the disease due to its ability to modulate epithelial function [8,9]. At present, fibroblasts and stromal changes occurring in CF are very poorly investigated. Huaux et al. showed lung fibroblasts have a role in CF [10] but they used the mouse model which is not considered to be fully representative of the human lung pathology [11]. Furthermore, the mechanisms underlying lung tissue remodeling in CF [12,13] are still unclear and there is not an in vitro model able to replicate connective tissue dysfunction in CF. In this scenario, we propose a 3D cystic fibrosis stroma model and deeply investigate its morphological features and molecular profile. This so-called 3D cystic fibrosis connective airway tissue equivalent (CF-CAT), developed by using a bottom-up tissue engineering strategy [14], was composed by primary lung cystic fibrosis fibroblasts embedded in their own ECM. For the first time, here we show the transcriptomic landscape of CF human lung fibroblast (CF-HLF) in comparison with normal human lung fibroblasts (N-HLF), evidencing differences that could offer a new perspective for therapeutic applications. We observed that CF-HLF in CF-CAT configuration over-expressed fibrotic markers and, more interestingly, up-regulated genes involved in the interaction with epithelial cells and inflammatory response, which are both key events in the progression of cystic fibrosis in vivo. Therefore, we strongly believe that the CF-CAT is a promising model to investigate stromal alterations and epithelial/stromal crosstalk in CF and, in the next future, to build up full-thickness organotypic model. In addition, our 3D model may be exploited as an in vitro human model to evaluate the patient-specific disease state and the efficacy of therapeutics targeting not only the epithelium but also the underlying connective tissue.

## 2. Materials and Methods

### 2.1. Cell Source

Normal/cystic fibrosis human lung fibroblast were purchased by the Lonza (N-HLF CC-2512 and CF-HLF CC-194843, respectively; EuroClone S.p.A. Milan, Italy). Both CF-HLF and N-HLF were extracted from the lung of donors belonging to the Caucasian population. Specifically, we used the lots 0000669504 and 0000548315 coming from male patients respectively 47 and 52 years old for the N-HLF CC-2512 code and the lot 9F3124 coming from a 45 years old female patient affected by cystic fibrosis for the CF-HLF CC-194843 code. The company Lonza guarantees they are negative for von Willebrand Factor Expression/Factor VIII, cytokeratin 18 and 19. In the case of alpha smooth muscle actin, cells are allowed to be up to 5% positive. Cell lots expressing more than this value do not pass their quality control and therefore are not be available for purchase. The information regarding the specific mutation of CFTR of the cystic fibrosis donor is not available from the company. Cells were cultured in enriched MEM: Minimum Essential Medium (Sigma Aldrich S.r.l. Milan, Italy) supplemented with 20%FBS (Sigma-Aldrich), 2% of Non-Essential Aminoacids (EuroClone), 1% of L-Glutamine (Microtech, Microgem Naples, Italy) and 1% of penicillin/streptomycin (Sigma Aldrich), until passage 7.

### 2.2. Evaluation of Endogenous Collagen Production

100% confluent fibroblast layers on fluorodish were treated for 10 days with Ascorbic Acid (2-O-α-D-Glucopyranosyl-L-Ascorbic-Acid; Final concentration: 0.5 mM, TCI Europe, Zwijndrecht, Belgium). The presence of collagen was monitored using multiphoton analysis as described in the following.

### 2.3. Micro-Scaffold Production

Gelatin porous micro-scaffolds were prepared according to a modified double emulsion protocol (O/W/O) and following crosslinked with 4% glyceraldehyde (Sigma Aldrich) and sterilized with 100% Ethanol for 24 h at Room Temperature (RT) [15,16].

### 2.4. Micro-Tissues (µTPs) Production

About 10 N-HLF/CF-HLF-CF P4/6 were seeded for micro-scaffold into spinner flask bioreactor (Integra). Cells were cultured in continuous agitation (30 rpm) as previously described [17]. The culture medium was enriched MEM plus Ascorbic Acid. Normal airway µTPs (NA-µTPs) and cystic fibrosis µTPs (CF-µTPs) were cultured for 18 days before the phase of assembly in order to guarantee the initial collagen synthesis. NA-µTPs and CF-µTPs were sampled during the culture period in order to monitor cell growth and the synthesis of elements of the extracellular matrix. Micro-tissue diameter was quantified using optical images and Image J measurement plugin. At different time points, the number of scaffolds was counted into a Nunc dish and, following, micro-tissues were digested using trypsin/collagenase type A (Roche, Final concentration 2.5 mg/mL, Merck Life Science S.r.l., Milan, Italy) in order to count cell numbers per micro-scaffold by using the Neu Bauer chamber.

### 2.5. Connective Airway Tissue (CAT) Production

The connective airway tissue (CAT) was engineered by using a previously described bottom-up approach [17,18]. NA-μTPs/CF-μTPs were transferred from the spinner flask to a sandwich-like maturation chamber in order to allow their molding in disc-shaped construct (usually 1 mm in thickness and 10 mm in diameter but also different shapes and dimensions). The chamber was placed on the bottom of a spinner flask (Bellco Vineland, NJ, USA) and completely surrounded by culture medium. The tissues were cultured in dynamic condition at 60 rpm. The culture medium was enriched MEM plus Ascorbic Acid. After 3/4 weeks of culture the maturation chamber was opened and normal airway connective tissue (NA-CAT) and cystic fibrosis connective tissue (CF-CAT) were collected.

### 2.6. Fluorescent Tissue Staining

Samples were fixed in 4% Paraformaldehyde (Sigma Aldrich) for 30 min at RT and washed in PBS1X. They were then permeabilized using 0.1% Triton (Sigma Aldrich) in PBS1X for 5 min at RT, washed in PBS1X and blocked in 1%BSA (Sigma Aldrich) for 1 h at RT. Afterwards samples were stained with Alexa Fluor 488-Phalloidin (Invitrogen, 1:200, Life Technologies Monza, Italy) for 45 min at RT and DAPI (Sigma Aldrich, 1:10.000) for 20 min at RT.

### 2.7. Multiphoton Analysis

For second harmonic generation (SHG), imaging samples were investigated by confocal microscopy (TCS SP5 II Leica) combined with MPM where the NIR femtosecond laser beam was derived from a tunable compact mode-locked titanium: sapphire laser (Chamaleon Compact OPO-Vis, Coherent). Two-photon excited fluorescence was used to induce SHG and obtain high-resolution images of unstained collagen structures. The samples were observed by using λ_ex_ = 840 nm (two photons) and λ_em_ = 415–425 nm. The SHG images were acquired with a resolution of 12 bit, 1024 × 1024 pixel by using a 25× water immersion objective (HCX IRAPO L 25.0X0.95 Water, n.a. 0.95).

### 2.8. Collagen Fraction Quantification

In order to quantify the collagen fraction, SHG images were analyzed by using the ImageJ software [19]. Regions of interest (ROIs) in the stroma compartment were chosen by excluding the signal of the micro-scaffold. The Collagen Fraction (%) Equation (1) was defined as the percentage of the ratio between bright pixels to total pixels in each selected ROI.

(1) $$Collagen\;Fraction\left(\%\right)=\frac{Nc\;}{Nb+Nc}*100$$

Nc and Nb represent the number of pixels from the collagenous and non-collagenous portion, respectively.

### 2.9. Gray-Level Co-Occurrence Matrix Analysis: Correlation

The gray level co-occurrence matrix (GLCM) analysis was performed using the ImageJ plugin Texture on SHG images as previously described [19]. The correlation was calculated for distances ranging from 1 to 100 pixels in the horizontal and vertical direction of each optical section. In such spatial windows the distance at which the correlation functions fall off represents the correlation length of the texture. Correlation length was obtained by fitting data with an exponential low.

### 2.10. Immunofluorescence on Tissue Sections

Samples were fixed in Formalin 10% (Sigma Aldrich) for 1 h at RT and washed in PBS1X (Sigma). They were dehydrated in ethanol from 75% to 100% and treated with Xylene (Romil) before the inclusion in paraffin. Tissue slices 7µm thick were cut using a microtome (Thermo Scientific) and then deparaffinized using xylene. Sections were hydrated in ethanol from 100% to 75% and washed in water, Triton 0.2%, and PBS1X. In order to release the epitopes from paraffin, for all the antibodies unless Hyaluronic Acid, heat mediated citrate buffer (pH = 6, Thermo Scientific, Life Technologies Monza, Italy, Europe) unmasking was performed. To release the epitopes for the detection of Hyaluronic Acid an enzymatic unmasking was performed rinsing the slices in a solution with CaCl2 (Merck) and Trypsin (Difco, Life Technologies Monza, Italy) at a final concentration of 0.01% in PBS1X (pH 7.8) for 20 min at 37 °C. Following, the sections were washed in PBS1X, blocked using 6% BSA, 5% FBS, 20 Mm MgCl_2_ and 0.02% Tween20 in PBS1X for 2 h at RT and incubated with the primary antibodies over night at 4 °C in a wet environment. Table 1 indicates primary antibodies used for immunofluorescence (Abcam, Cambridge, UK).

The morning after samples were washed in PBS1X and the secondary antibodies (Alexa Fluor, Table 2) diluted in PBS1X were added for 1 h and half at RT. Nuclei were stained with DAPI and samples were investigated by Confocal Leica TCS SP5 II.

### 2.11. Protein Quantification

Ten images per sample were acquired in different points of the 3D tissue and analyzed by using ImageJ. In order to estimate the quantity of protein per cell, the area relative to the fluorescent protein signal was thresholded, quantified (in µm^2^), and divided for the number of cells obtained by counting cell nuclei stained by DAPI with “Analyze particles” of Image J.

### 2.12. Mechanical Properties

The mechanical properties of CAT (diameter 10 mm and thickness 1 mm) were analyzed by means of Piuma Nano-Indenter (Optics). It is a displacement-controlled nano-indenter machine including a controller, an optical fiber and a spherical probe. The probe is attached to a spring cantilever that is connected to the end of optical fiber in order to measure the cantilever deflection. Each sample was indented using a cantilever with a stiffness of 4.53 Nm^−1^. The indentation depth was about 10 µm during each indentation test performed in 5 different points of each sample. The tip radius was 53.5 μm and the connected optical fiber measured the cantilever deflection during indentation. Based on the indentation of surfaces using probes with a well-defined geometry, the elastic and viscoelastic constants of examined materials can be determined by relating indenter geometry and measured load and displacement to stress and deformation. The analysis of spherical nano-indentation data is generically based on the Hertz model, assuming a linear elastic and isotropic material response. In order to characterize material viscoelastic and anisotropic proprieties like biological tissue and to study the analysis of spherical nano-indentation data, we used the Hertz model and a variant of the nano-epsilon method (M) [20]. The elastic modulus (E) Equation (2) was obtained as ratio between the indentation stress (σ *ind)* Equation (3) and strain *(ε ind)* Equation (4).

(2) $$E=\frac{\sigma \;ind}{\varepsilon \;ind}$$

(3) $$\sigma \;ind=\frac{P}{R\sqrt{hR}}$$

(4) $$\varepsilon \;ind=\frac{4h}{3R\left(1-v^{2}\right)}$$

where P was the load, R the radius of spherical indenter tip, and h the penetration depth.

### 2.13. RNA Extraction

3D Samples were collected, washed and digested in Trizol (Thermo Fisher Scientific S.p.A. Rodano (MI), Italy, Europe) on a vortex by using a stainless-steel bead and then passing the solution into a syringe needle. After homogenization, samples were treated with 200 µL of chloroform, vortexed, and centrifuged at 12,000 rcf for 10 min. 500 µL of isopropanol was added to the upper phase. Samples were incubated for 10 min at RT and centrifuged at 12,000 rcf for 10 min. After the removal of the supernatant, pellets were washed with 1ml of EtOH 75%, centrifuged for 10 min at 7600 rcf, and then resuspended in 100 μL of RNase-free water. To perform RNA precipitation, 100 μL of acid phenol-chloroform was added to the sample. After a centrifugation of 15minutes at maximum speed, 9 μL NaAC (3M) and 250 μL EtOH 100% were added to the upper phase. Samples were vortexed and incubated at −80 °C for 1 h. Then, tubes were centrifuged for 15 min at maximum speed (4 °C). After removing the supernatant, the pellet was resuspended in RNase-free water. To further clean the RNA sample from collagen debris the solution was transferred into the column of the High Pure RNA Tissue Kit (Roche), centrifugated at the maximum speed. RNA was eluted in 20 µL of RNase-free water. Similarly, 2D samples were extracted by using the same protocol, excluding the phase of tissue homogenization.

### 2.14. QuantSeq 3’ mRNA Sequencing Library Preparation

Preparation of libraries was performed with a total of 100 ng of RNA from each sample using QuantSeq 3’mRNA-Seq Library prep kit (Lexogen, Vienna, Austria) according to manufacturer’s instructions. Total RNA was quantified using the Qubit 2.0 fluorimetric Assay (Thermo Fisher Scientific). Libraries were prepared from 100ng of total RNA using the QuantSeq 3’ mRNA-Seq Library Prep Kit FWD for Illumina (Lexogen GmbH). Quality of libraries was assessed by using screen tape High sensitivity DNA D1000 (Agilent Technologies). Libraries were sequenced on a NovaSeq 6000 sequencing system using an S1, 100 cycles flow cell (Illumina Inc.). Amplified fragmented cDNA of 300 bp in size were sequenced in single-end mode with a read length of 100 bp.

Illumina NovaSeq base call (BCL) files are converted in fastq file through bcl2fastq [http://emea.support.illumina.com/content/dam/illuminasupport/documents/documentation/software_documentation/bcl2fastq/bcl2fastq2-v2-20-software-guide-15051736-03.pdf] (v2.20.0.422).

### 2.15. QuantSeq 3’ mRNA Sequencing Data Processing and Analysis

For analysis, sequence reads were trimmed using bbduk software (https://jgi.doe.gov/data-and-tools/bbtools/bb-tools-user-guide/usage-guide/) (bbmap suite 37.31) to remove adapter sequences, poly-A tails, and low-quality end bases (regions with average quality below 6). Alignment was performed with STAR 2.6.0a3 [21] on hg38 reference assembly obtained from cellRanger website (https://support.10xgenomics.com/single-cell-gene-expression/software/release notes/build#GRCh38_3.0.0; Ensembl assembly release 93). Expression levels of genes were determined with htseq-count [22] using Gencode/Ensembl gene model [23]. We have filtered out all genes having < 1 cpm in less than n_min samples and Perc MM reads >20% simultaneously. Differential expression analysis was performed using edgeR [24], a statistical package based on generalized linear models, suitable for multifactorial experiments. The threshold for statistical significance chosen was False Discovery Rate (FDR) < 0.05: in detail, 2005 genes were differentially expressed (1179 genes induced and 826 inhibited) in the 2D dataset (GSE141535), while 1505 genes were differentially expressed (968 genes induced and 537 inhibited) in the 3D dataset (GSE141536). The comparison of the two datasets is represented in the venn diagram in the paragraph 3.3 of the Results and the lists of genes obtained are included in the Supplementary Table S3: 1406 transcripts were specifically regulated in the 2D condition (779 up-regulated and 627 down-regulated), 906 transcripts were specifically regulated in the 3D condition (554 up-regulated and 352 down-regulated), 559 differentially expressed genes (DEGs) were commonly regulated (381 up-regulated and 166 down-regulated in both datasets and 52 regulated in opposite manner). Gene Ontology (GOEA) and Functional Annotation Clustering analyses were performed using DAVID Bioinformatic Resources [25,26] restricting the output to Biological Process terms (BP_FAT). Cellular Compartment terms (CC_FAT) and Molecular Function terms (MF_FAT). The ‘Kyoto Encyclopedia of Genes and Genomes’ (KEGG Pathway) analyses [27,28,29] was also performed. The threshold for statistical significance of GOEA was FDR < 0.1 and Enrichment Score ≥1.5, while for the KEGG Pathway analyses was FDR < 0.1. The GOEA was performed on the induced genes in the two datasets separately (Supplementary Table S1, and S2) and after the comparison on the commonly up-regulated and on the specifically up-regulated gene lists (Supplementary Table S4). The additional threshold of logFC >2 for induced and logFC < −2 was used for the list of DEGs obtained in the two datasets separately. In details the DEGs induced into the 3D dataset were 487 (Supplementary Table S1, GSE141536), while the DEGs induced into the 2D dataset were 459 (Supplementary Table S2, GSE141535). The results of the GOEA performed on the 554 transcripts specifically up-regulated in the 3D dataset after the comparison with the 2D were reported in the paragraph 3.3 of the Results and in the Supplementary Table S4.

### 2.16. Accession Codes

The whole set of results is available in the GEO database as SuperSeries code GSE141537. The title of the SuperSeries is “Transcriptome profile of Primary Human Lung Fibroblasts (Cystic Fibrosis vs. Non-Cystic Fibrosis)”. In details: 1) GSE141535 refers to expression data from Primary Human Lung Fibroblasts (Cystic Fibrosis vs. Non-Cystic Fibrosis) in 2D condition; 2) GSE141536 refers to expression data from Primary Human Lung Fibroblasts (Cystic Fibrosis vs. Non-Cystic Fibrosis) arranged in a 3D model of the connective airway tissue.

### 2.17. The Accession for the Reviewer

The following secure token has been created to allow review of record GSE141537 while it remains in private status: cfuzygkydlmdfor.

### 2.18. Statistical Analysis

Morphological experiments were performed in triplicate. Data were expressed as mean ± SD. Differences between groups were determined using the statistic test ANOVA Tukey HSD test. Significance between groups was established with a ^*^p value <0.05 or **p value <0.01.

Transcriptomic analyses were performed in triplicate (3D) or quadruplicate (2D). Only DEG with False Discovery Rate (FDR) <0.05 were considered as statistically relevant.

## 3. Results

### 3.1. Bioengineering Bottom-Up Approach Leads to Connective Airway Tissue Equivalent Production

**Figure 1:**
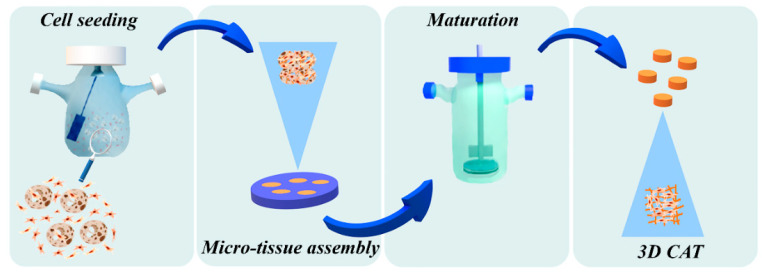
Bio-engineered process to develop the cystic fibrosis (CF) connective airway tissue (CAT): Cystic fibrosis human lung fibroblasts (CF-HLF) were seeded on porous gelatin micro-scaffolds into spinner flasks bioreactor; 18 days after the seeding of the fibroblasts cystic fibrosis micro-tissues (CF-µTPs) were retrieved and assembled into maturation chambers, after 3 weeks it was possible to obtain the final connective airway tissue (CAT), featured by the presence of fibroblasts embedded in their own matrix. The same process, but starting from normal human lung fibroblasts (N-HLF), was used to obtain the normal connective airway tissue (NA-CAT) as experimental control.

**Figure 2:**
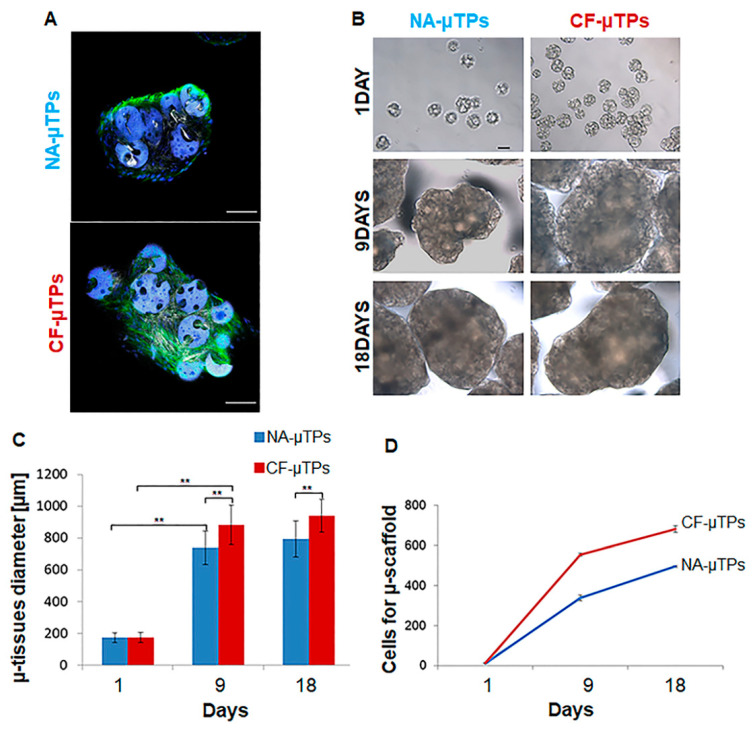
µTPs morphological characterization. (**A**) Representative images of normal and cystic fibrosis micro-tissues (NA-µTPs and CF-µTPs) showing the nuclei of the cells marked in blue by DAPI, the actin cytoskeleton in green by 488-Phalloidin and the collagen in gray by second harmonic generation signal (SHG), scale bar 100 µm; (**B**) optical microscope images of NA-µTPs and CF-µTPs 1, 9, and 18 days after the seeding of the fibroblasts on the micro-scaffolds, scale bar 100 µm; (**C**) Histogram of NA-µTPs (blue) and CF-µTPs diameter (red) over time (** *p* value <0.01), data are showed as Mean ± Standard Deviation; (**D**) Graphic showing the number of cells per micro-scaffold over time in n NA-µTPs (blue) and CF-µTPs (red).

**Figure 3:**
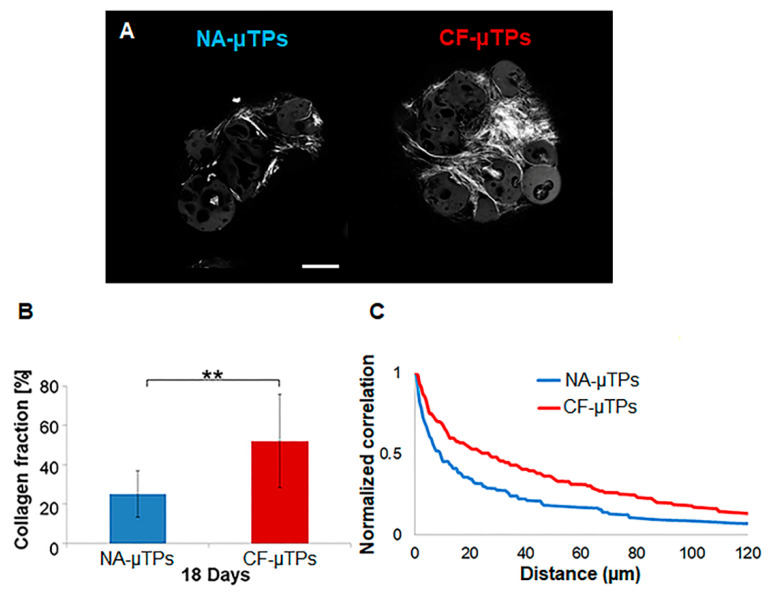
µTPs morphological characterization: (**A**) Second harmonic generation (SHG) images of collagen fibers (shiny gray) in normal and cystic fibrosis micro-tissues (NA-µTPs and CF-µTPs); (**B**) Diagram of the collagen fraction (%) in NA-µTPs (bleu) and CF-µTPs (red), data are showed as Mean ± Standard Deviation; (**C**) Graphic of the normalized correlation obtained analyzing SHG images of NA-µTPs (blue) and CF-µTPs (red).

Firstly, we evaluated the capability of both normal and cystic fibrosis human lung fibroblasts (N-HLF and CF-HLF) to produce endogenous collagen in vitro by stimulating 100% confluent cell layer with ascorbic acid for 1 week. Supplementary Figure S1 shows the de novo synthetized matrix in gray thanks to the Second Harmonic Generation (SHG) signal of endogenous collagen fibers. Then, we obtained lung connective airway tissue (CAT) by slightly modifying a previously established tissue engineering bottom-up approach [16] as illustrated in Figure 1. Both N-HLF and CF-HLF were able to adhere on porous gelatin micro-scaffolds, to proliferate and synthetize elements of the Extracellular Matrix (ECM) in dynamic culture conditions. Cell–cell and cell–matrix interactions over time enabled the formation of Normal and CF micro-tissues (NA-µTPs and CF-µTPs). Both micro-tissues featured the presence of the cells embedded into the endogenous collagen matrix as showed in Figure 2A. Both NA-µTPs and CF-µTPs increased their size over time but CF-µTPs were larger than NA-µTPs at each time point (optical image in Figure 2B and graphic in 2C). The number of cells per µ-scaffold demonstrated that CF lung fibroblasts proliferated faster than normal ones (Figure 2D). Moreover, CF fibroblasts produced a higher amount of collagen as visible in the SHG images (Figure 3A) and by the analysis of the collagen fraction (Figure 3B). The increased number of cells and the amount of collagen agrees with the rise in size of CF-µTPs compared to NA-µTPs. Collagen network morphometry was then investigated by analyzing SHG images with the correlation feature of the Gray Level Co-occurrence matrix (GLCM) [30,31,32]. The correlation curve of CF-µTPs decayed slower than the NA-µTPs one (Figure 3C), indicating a more disorganized collagen network in the disease model [33]. Moreover, the correlation length of CF-µTPs was significantly higher than NA-µTPs (Table 3), demonstrating the presence of thicker collagen fibers in the disease model [34].

### 3.2. Cystic Fibrosis Connective Airway Tissues Overexpress Extracellular Matrix (ECM) Macromolecules Synthesis and Assembling

**Figure 4:**
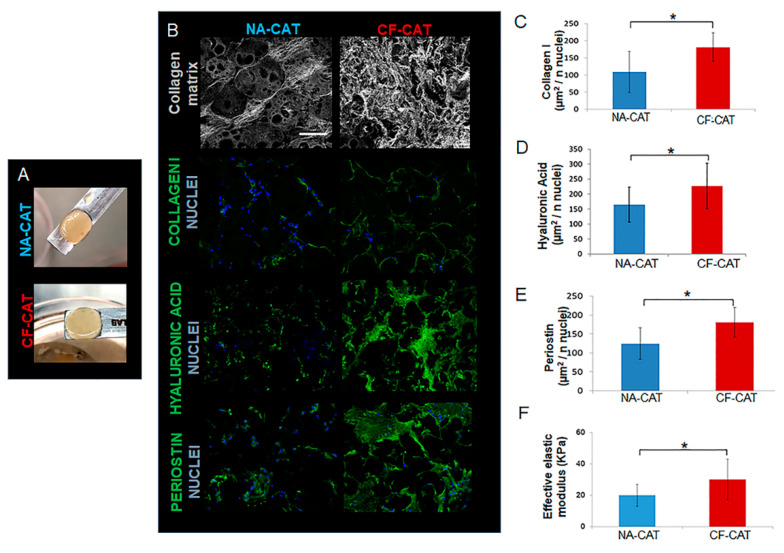
CAT morphological analysis. (**A**) Macroscopic image of a representative normal and cystic fibrosis connective airway tissue (NA-CAT and CF-CAT); (**B**) Second harmonic generation (SHG) images of collagen fibers in gray and immunofluorescence showing the nuclei of the cells in blue marked by DAPI and in green the signal of Collagen Type I/Hyaluronic Acid and Periostin; scale bar 100 µm; (**C**) Diagram of the quantity of collagen (µm^2^) type I per cell (nuclei) NA-CAT (blue) and CF-CAT (red); (**D**) Diagram of the quantity of Hyaluronic Acid (µm^2^) type I per cell (nuclei) NA-CAT(blue) and CF-CAT (red); (**E**) Diagram of the quantity of Periostin (µm^2^) type I per cell (nuclei) NA-CAT(blue) and CF-CAT(red); (**F**) Diagram of the effective elastic modulus of the NA-CAT(blue) and CF-CAT(red). In all the diagrams data are showed as Mean ± Standard Deviation *p value <0.05.

In the second step of the process, the “biosintering” of NA-µTPs or CF-µTPs, meaning the biological fusion of the micro-tissue units of the same type [18], was induced to obtain the Normal (NA-CAT) or the CF Connective Airway tissue (CF-CAT) (Figure 1). Specifically, the “biosintering” of CF-µTPs led to the formation of the CF-CAT whose morphological features were compared with the NA-CAT, likewise obtained by assembling NA-µTPs. The macroscopic appearance of the CF-CAT and NA-CAT was very similar, as shown in Figure 4A. However, multiphoton microscopy and immunotypization of both models showed that the CF-CAT is characterized by a higher amount of fibrillar collagen, collagen type I, hyaluronic acid and periostin (Figure 4B–E). Fibrillar collagen was observed by exploiting SHG imaging. The gray signal in the images in Figure 4B highlights the higher quantity of collagen and the more chaotic architecture of the network in the CF-CAT compared to NA-CAT, as already found in micro-tissues. Collagen type I is the main structural protein of the connective tissues responsible for their mechanical properties [35]. Hyaluronic Acid is a large unsulfated glycosaminoglycan, important for tissue repair and homeostasis [36]. Periostin is a matricellular protein with non-structural function, involved in tissue development, repair and remodeling [37]. On the other hand, periostin is involved in different pathological processes such as lung fibrosis [37]. It is able to induce collagen fibrillogenesis and influence tissue mechanical properties [38]. Indeed, our results on mechanical characterization by nano-indentation test (Figure 4F) revealed a higher effective elastic modulus in the CF-CAT than NA-CAT, typical of fibrotic tissues [39].

### 3.3. Transcriptomics Analysis Reveals a Fundamental Role of the 3D Stromal Environment in Up-Regulating Genes Involved in the Epithelial Morphogenesis and Inflammatory Response

**Figure 5:**
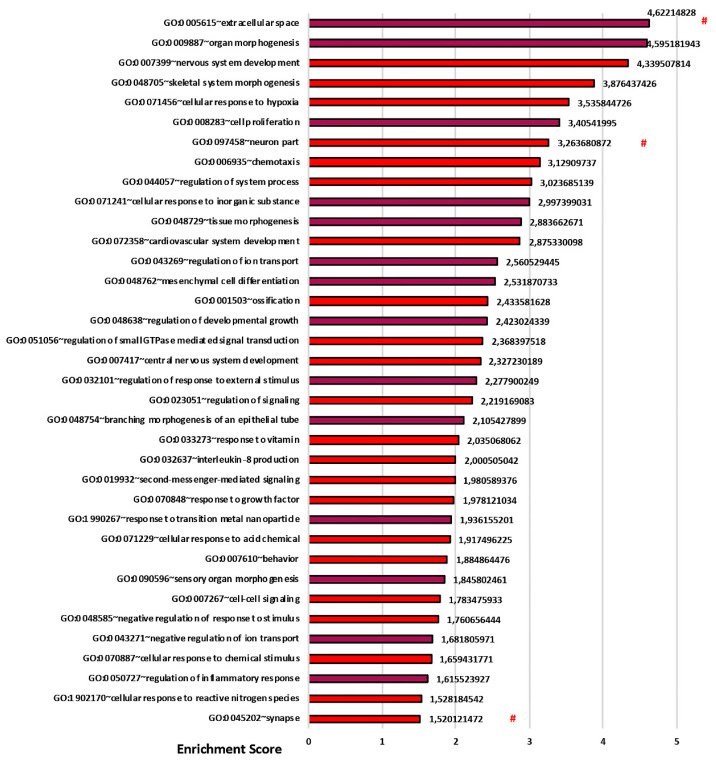
3D CAT molecular analysis (CF-HLF vs. N-HLF). Biological process (BP) and cellular component (CC) significant terms in which the DEG (differentially expressed genes) induced into the 3D dataset are mainly enriched (GSE141536). The threshold of induction plotted is logFC > 2 (487 DEG). # are CC terms. Dark Red terms are the mainly interested induced terms found in our study.

To further investigate the effect of the molecular signature underlying the morphological differences observed between CF and Normal fibroblasts (CF-HLF and N-HLF), we performed an unbiased RNA-seq analysis of the cells arranged in 2D or in 3D (CAT) configurations (GSE141537). The transcriptomic analysis likewise confirmed the differences observed between CF-HLF and N-HLF and highlighted a fundamental role of the 3D environment in replicating tissue physio/pathology in vitro. Moreover, as both CF-HLF and N-HLF did not express CFTR, the observed differences between CF-HLF and N-HLF are not associated with CFTR dysfunction in vitro but with other factors probably derived from the patient specific in vivo lung condition at the moment of the biopsy from which the primary cells were extracted. Both 2D and 3D conditions were analyzed by comparing the transcriptome of CF-HLF to the transcriptome of N-HLF used as control. In both conditions, we observed a significant alteration of the transcriptome: in details, we found 2005 and 1505 differentially expressed genes (DEGs) in the 2D and in the 3D transcriptomes, respectively. To investigate the effect of the 2D or the 3D condition on the gene expression profiles and to identify the most deregulated pathways, we performed gene ontology enrichment analysis (GOEA) [25] on the two datasets. We observed the term “Extracellular space” (GO:0005615) comprising 81 genes, as the most significant according to GOEA in 3D (Figure 5 and Supplementary Table S1). Similarly, in the 2D dataset the most enriched term was “Extracellular region part” (GO:0044421) (Supplementary Figure S2 and Supplementary Table S2). Moreover, in agreement with morphological results, CF-HLF over-expressed genes related to the “Extracellular space” (GO:0005615), “Cell proliferation” (GO:0008283) and “Tissue morphogenesis” (GO:00487298) when compared to N-HLF in 3D (Figure 5 and Supplementary Table S1). We observed a set of genes over-expressed by CF-HLF in CF-CAT which included: hyaluronan synthase 2 (HAS-2), indicating a positive correlation between gene expression and the quantity of hyaluronic acid observed by immunofluorescence; TWIST1 and TGFβ3, positive regulators of periostin synthesis [40,41], and WNT2, SFRP2, and RSPO2 in the WNT pathway. The latter suggests an aberrant activation of the WNT pathway in lung fibroblasts of patients with CF, similarly as that observed during lung fibrosis [42,43]. Other genes activated in CF-HLF as in lung fibrosis were: TBX4 [44] and ITGA8 [45]. HHIP and CHI3L1 were both over-expressed in CF as well as in chronic obstructive pulmonary disease (COPD) and asthma [46,47] (Table 4).

**Figure 6:**
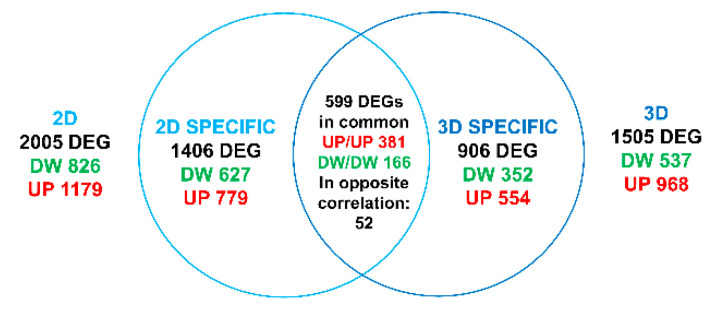
Comparison between the 2D and 3D datasets: Venn diagram of transcriptomic results comparing cystic fibrosis vs. normal human lung fibroblasts (CF-HLF vs. N-HLF) in 2D and 3D culture conditions.

**Figure 7:**
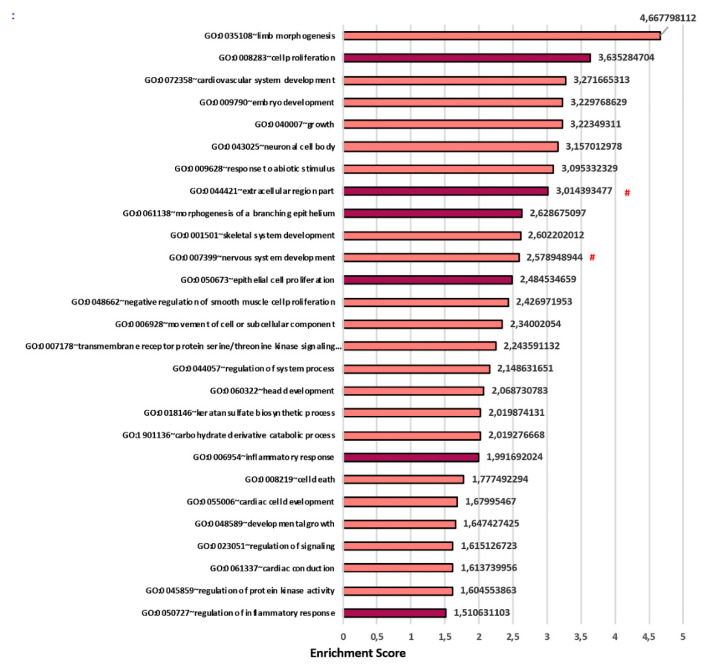
Specific 3D CAT molecular analysis (CF-HLF vs. N-HLF): Biological process (BP) and cellular component (CC) significant terms in which the DEG (differentially expressed genes) specifically induced (554) into the 3D dataset are mainly enriched. # are CC terms. Dark Red terms are the mainly interested induced terms found in our study.

We then compared the two datasets (2D and 3D), each one normalized on its own control, as represented in the Venn diagram (Figure 6). The genes of the datasets in comparison are specifically listed in Supplementary Table S3 and the GOEA is showed in Supplementary Table S4. This analysis allowed to isolate the genes whose regulation was either dependent or independent of the two different culture conditions, and thus to isolate transcripts specifically regulated in 2D (1406 in total divided in 779 up-regulated and 627 down-regulated), and specifically regulated in 3D (906 in total divided in 554 up-regulated and 352 down-regulated). Furthermore, we isolated 559 DEGs commonly regulated in both 2D and 3D: 381 up-regulated and 166 down-regulated in both datasets, respectively, whereas 52 were regulated in an opposite manner. Considering the 381 DEGs commonly induced, GOEA highlighted terms such as “Organ morphogenesis” (GO:0009887), “Cell proliferation” (GO:0008283), “Tissue morphogenesis” (GO: 0048729), and “Response to external stimulus” (GO: 0009605) (Supplementary Figure S3). This result strongly suggests that CF-HLF retained an activated state in vitro both in 2D and 3D. The 2D specific analysis showed the enrichment of the GO terms “Epithelial cell proliferation” (GO:0050673), “Regulation of epithelial to mesenchymal transition” (GO:0010717), “Canonical Wnt signaling pathway” (GO:0060070) and “Metal ion binding” (GO:0046872). The latter was not enriched in the 3D specific induced dataset (Supplementary Table S4 and Supplementary Figure S4). We hypothesize that the specific upregulation of the GO term “Metal ion binding” in the 2D culture may be related to this particular condition in which all cells are in direct contact with the culture medium that is rich in ions affecting cell behavior in vitro [48]. Instead, cells in the 3D model, particularly those present in the inner regions of the sample, may sense a different environment as a result of modifications introduced by ECM itself. Among the genes up-regulated by CF-HLF (Table 4): TGFB3, SFRP2, RSPO2, ITGA8, and CHI3L1 were specifically upregulated only in the 3D culture condition and HAS-2 was over-expressed in 3D and down-regulated in 2D. Interestingly, in the GOEA performed on the 3D transcriptome highlighted terms such as “Morphogenesis of a branching epithelium” (GO: 0061138), “Inflammatory response” (GO: 0006954) and “Regulation of inflammatory response” (GO: 0050727) (Figure 7 and Supplementary Table S4). These results suggest that only the 3D environment of the CF-CAT was able to induce the expression of genes involved in these key events occurring during the progression of cystic fibrosis in vivo.

## 4. Discussion

Traditionally, in vitro study of CF lung disease has focused on the study of airway epithelial cells carrying CFTR mutations. Consistent with the role of stromal environment in several diseases affecting epithelium [49,50,51], here we show that the pulmonary CF stroma has non-negligible abnormalities that in vivo may have a significant impact on the progression of the lung disease. Indeed, our results demonstrated morphological and transcriptomic differences existing between primary CF-HLF and N-HLF and the importance of the 3D environment to highlight such features in vitro. In this work, we developed a 3D bio-engineered model of CF connective airway tissue (CF-CAT) and compared its characteristics with the normal counterpart (NA-CAT). Specifically, the CF-CAT was achieved in vitro by using a bottom up approach (Figure 1) and it featured the presence of fibroblasts embedded in their own native ECM. By using the same approach, our group already demonstrated the possibility of recapitulating in vitro the physio-pathological environment of different tissues in terms of composition and features of the organ-specific ECM, as well as interaction between the epithelial and stromal compartments [9,52,53]. CF arises from CFTR mutation in epithelial cells, but the severe complications of this pathology involve the entire pulmonary tissue and dramatically compromise lung functions. Indeed, mucus plugging, bacterial infection [2], inflammatory response [54,55], and tissue remodeling [12,56] are all central features of CF and represent putative therapeutic targets. In this context, our model uniquely recapitulated lung tissue remodeling and fibrosis occurring during CF. In fact, lung fibroblasts coming from patients with CF displayed an activated and fibrotic phenotype with an increase in cell proliferation (Figure 2D), production of ECM elements (Figure 3A,B, Figure 4B–E), collagen fiber thickness, and aberrant organization of the network (Figure 3C, Table 3, Figure 4B). Among the ECM proteins we found that CF-HLF produced higher quantity of collagen I, hyaluronic acid, and periostin. The latter has been recognized as marker of lung fibrosis and inflammation [37]. Moreover, periostin was reported to have a role in collagen synthesis [38], assembly, and organization in different pathological conditions [57]. It binds other proteins of the ECM, and the bone morphogenic protein 1 (BMP-1), catalyzing the crosslinking of collagen fibers and increasing tissue stiffness [58]. At the same time, periostin activates HAS-2 function and hyaluronic acid synthesis [59]. The higher quantity of hyaluronic acid and the overexpression of HAS-2 we found in the CF-CAT were also markers of severe fibrosis [60]. The presence of important abnormalities in CF-HLF behavior, despite the absence of CFTR expression, is probably a consequence of the in vivo activation of the cells during the chronic pathology. It appears that CF-HLF in vitro retain epigenetic changes resulting from the cascade of events that start from CFTR loss of function and lead to severe inflammation in vivo. This hypothesis relies on the fact that primary cells in vitro can maintain an epigenetic memory related to the cell state in vivo [61]. Moreover, the presence of a physiological 3D ECM environment represents a clinically relevant model to targeted epigenetic therapies [62,63]. Indeed, CF is a monogenic disease caused by CFTR mutation in the epithelium. Nevertheless, the consequences of the epithelial dysfunction affect the entire lung tissue with increased susceptibility to infection, reiterative stresses and overproduction of reactive oxygen species. These events are known to be responsible for the establishment of unique DNA methylation profiles which modulate disease phenotype and severity. Altered methylation levels were already found in nasal epithelial and blood cell samples from CF patients [64,65]. We suppose that the altered lung environment in CF could affect the DNA methylation profile of stromal fibroblasts as well. This may happen similarly as shown for lung fibrosis, where the loss of epithelial integrity and aging associated events trigger epigenomic changes and stochastic profibrotic methylation drift leading the development of the pathology [66,67]. As of now, we did not evaluate the epigenetic profile of the cells, but we think it will be an interesting point to examine in depth. In this work we focused on the transcriptomic profile of CF-HLF. Our results demonstrated that CF-HLF up-regulated genes for the production of elements in the extracellular space compartment and for the biological processes of tissue/organ morphogenesis and cell proliferation (Figure 6). At the same time, they overexpressed genes of the WNT pathway, TBX4, and ITGA8, which have been demonstrated to play a part in lung fibrosis [42,43,44,45]. Moreover, although we confirmed that lung fibroblasts do not express CFTR, we evaluated CF-HLF up-regulated the GO terms “Regulation of ion transport” (GO:0043269) and “Negative regulation of ion transport” (GO:0043271). We hypothesize that such overexpression could be a consequence of the in vivo lack of CFTR function in epithelial cells. On the other hand, transcriptomic analysis showed that CF-HLF upregulated genes involved in the biological processes of “Mesenchymal cell differentiation” (GO:0048762) (Figure 6), “Mesenchyme development” (GO:0060485) and “Mesenchymal cell proliferation” (GO:0010463) (Figure S2) suggesting CF-HLF may have the potential to activate the Epithelial to Mesenchymal Transition (EMT). Specifically, the upregulation of the GO Term “Mesenchymal cell differentiation” (GO:0048762) which comprises genes involved in the EMT (such as FAM83D, WNT2, MSX2, SFRP2, HAS2, TMEM100, AXIN2, BMP7, and TWIST1 showed in Supplementary Table S5), lead us to hypothesize that stromal lung fibroblasts in vivo may have a role in the induction of the EMT. This phenomenon has been well characterized in other lung diseases and it has recently associated with CF [5]. Interestingly, the process of EMT has been always recognized as a driving mechanism of lung fibrosis [5,68] but here we show that the activation of lung fibroblasts during fibrosis can, in turn, stimulate EMT. However, the upregulation of the aforementioned GO terms supported fibroblast-to-myofibroblast differentiation and myofibroblast biogenesis in our model, as typic of fibrotic states. Furthermore, we highlighted that the GO term “Morphogenesis of a branching epithelium” (GO:0061138) was enriched only in the 3D culture condition and not in 2D (Figure 7). Already in the past, it has been demonstrated that the presence of the stromal compartment is necessary for epithelial branching during the in vivo development of the lung [69]. Similarly, here we observed that the connective tissue has a central role in the morphogenesis of epithelial structures in a 3D in vitro environment. Moreover, our data show that the formation of branching epithelial structures can be altered in CF due to an increased stimulus coming from fibroblasts. We assume that such biological process can be involved in the dysregulation of submucosal gland morphology and function observed in CF. Indeed, these structures develop through a process of branching morphogenesis in vivo [70] and are hyperplasic and mucus occluded in the disease state [71,72]. Furthermore, submucosal glands alteration has been recognized as an important factor contributing to CF physiopathology [73]. Submucosal glands in vivo develop through a process of epithelial cell branching from the epithelium into the underlining connective tissue. Specifically, the process involves a first phase of placode formation in the epithelium followed by a phase of elongation and branching of the cells into the stroma and the final differentiation [70]. Previous works showed that the morphology and the function of submucosal grands are dramatically compromised in CF, most probably because a high percentage of epithelial cells in these glands should express CFTR in the normal condition. As consequence of CFTR defection, submucosal glands become hyperplasic and mucus occluded in CF [71,72]. In this scenario, the overexpression of the genes for the “Morphogenesis of a branching epithelium” in the CF-CAT suggests a role of the ECM in regulating the morphogenesis of epithelial structures in the disease state in vivo. On the other hand, the upregulation of the GO term “Morphogenesis of a branching epithelium” in the 3D CF model underlined that fibroblasts, even if cultured without epithelial cells, in such a physiological relevant in vitro environment were able to ‘store memory’ of the epithelial stromal crosstalk occurring in vivo. For this reason, we are confident that the CF-CAT could be used as ‘biological soil’ for the formation of submucosal glands in vitro and for the study of their dysfunction in cystic fibrosis in future studies. At the same time, CF-HLF overexpressed genes involved in the “Inflammatory response” (GO:0006954) also without inflammatory stimuli in vitro (Figure 7). Indeed, inflammation is a hallmark of CF due to the structural lung tissue change and the persistent bacterial colonization of the airways [54,55]. Our results demonstrated lung fibroblasts contribute to the amplified inflammatory response observed in CF patients [10]. Among the genes of the inflammatory response we identified the up-regulation of ILR6 in CF-CAT. This gene encodes a subunit of the interleukin 6 (IL6) receptor complex which is a potent pleiotropic cytokine playing an important role in the immune response. The IL6R can be transmembrane or soluble. The soluble form of the IL6R is known to increase the kind and number of cells targeted by IL6 through the mechanism of trans-signaling, able to mediate pro-inflammatory activities. For this reason, the IL6R is the receptor of a monoclonal antibody (tocilizumab) used for the treatment of several inflammatory diseases [74,75]. The IL-6R expression by activated fibroblasts was already demonstrated in other inflammatory states [76] and here we report its involvement in the cystic fibrosis lung stroma. In conclusion, the transcriptomic analysis highlighted the importance of the 3D microenvironment in the expression of genes involved in the crosstalk between the stroma and the epithelium, inflammation, and fibrosis. All these processes are implied in the evolution of the human pathology. In fact, stromal/epithelial crosstalk has consequences on both tissue morphogenesis and function/dysfunction, and inflammatory response and fibrosis are responsible for the occurrence of severe complications in patients with cystic fibrosis. Our work sheds light on the alterations of the connective airway tissue in CF which cannot be neglected for the study of this pathology and for the development of therapeutic strategies. Our findings demonstrated that the CF-CAT showed interesting features in common with lung fibrosis. In this regard, we believe that the 3D in vitro stroma lung model is able to highlight important alterations responsible for the loss of tissue function during the chronic pathology in vivo. In particular we believe that the CF-CAT recapitulates late stage of the human pathology, the fibrosis after infection and inflammations, which is difficult to treat in the clinic. In this context we identified higher amount of collagen type-I, hyaluronic acid, and periostin in the CF-CAT and the upregulation of several transcripts by CF-HLF (such as TGFβ3, HAS-2, TWIST1, WNT2, SFRP2, RSPO2, CHI3L1, TBX4, ITGA8, CHI3L1, HHIP). We argue that such markers could be the targets of novel therapeutic strategies aiming to rescue lung function in CF patients with lung fibrosis. Most probably, the connective tissue changes come from the epithelial damage and in turn impair epithelial function. Furthermore, we believe that the 3D CF-CAT model is suitable to replicate the patient specific state of the disease at the stroma level. For this study we chose to compare CF-HLF and N-HLF coming from patients having similar ages. Specifically, we used cells from adult donors: 45 for CF-HLF and 47/52 years old for N-HLF. Cells coming from younger patients are generally more active than older [77]. At the beginning of our study we performed some experiments with N-HLF from a 3 years old child. Our results showed that young fibroblasts were able to produce a high quantity of collagen, almost comparable to a CF old patient (Supplementary Figure S5). Unfortunately, we did not go ahead with this study because no CF-HLF vial from young donors was available from Lonza. We suppose that normal younger cells may respond to pathological insults in a more physiological way through the activation of mechanisms of the ECM remodeling able to preserve tissue homeostasis and function [78]. Furthermore, the age of the patient may reflect different pathological states and so different condition of the stroma (e.g. not fibrotic or fibrotic). To validate this hypothesis, in future studies we will directly extract fibroblasts from human biopsies with the aim to obtain a wider “age” range of connective airway tissues in vitro and compare their features with the corresponding primary biopsy. At last, we argue the CF-CAT may be used in the future as a feeder for the seeding of epithelial cells with the aim to obtain a unique full thickness model of CF and investigate the consequences of the epithelial stromal crosstalk in CF on a 3D reliable platform.

## 5. Conclusions

This work exploits a novel 3D in vitro model of the pulmonary stroma to demonstrate the morphological and molecular alterations in the cystic fibrosis airway connective tissue. The model was obtained by using primary cystic fibrosis lung fibroblasts and a bottom up approach of tissue engineering. Confocal, multiphoton, and RNA sequencing analysis revealed that cystic fibrosis lung fibroblasts over-express pro-fibrotic markers and produce an abundant and chaotic matrix compared to the normal condition. Moreover, they over-express genes involved in the communication with the epithelium and inflammatory response, thus demonstrating a pivotal role in the progression of the human pathology. For the first time, here we showed the transcriptomic profile of cystic fibrosis lung fibroblasts in comparison with normal fibroblasts and highlighted fundamental differences. We argue that such a study may provide novel food for thought for the development of novel therapeutic strategies targeting the stroma, besides underlining the role of the pulmonary stroma in cystic fibrosis.

## Figures and Tables

**Table 1:** List of the primary antibodies used for immunofluorescence.

Antibody	Code	Working Dilution	Unmasking
CollI (Rabbit)	ab34710	1:500	Heat mediated (citrate buffer)
Hyaluronic Acid (Sheep)	ab53842	1:50	Enzymatic (Trypsin)
Periostin (Rabbit)	ab14041	1:100	Heat mediated (citrate buffer

**Table 2:** List of the secondary antibodies used for immunofluorescence.

Secondary Antibody	Code	Working Dilution
488 Goat Anti-Rabbit	A11008	1:500
546 Donkey Anti-Sheep	A21098	1:500

**Table 3:** Correlation length analysis.

Sample	Correlation Length (λ)
**NA-µTPs**	62.6254 ± 16.23
**CF-µTPs**	135.88 ± 43.31

**Table 4:** Up-regulated genes from the 3D dataset (CF-HLF vs. N-HLF).

Gene Symbol	logFC_CF-HLF vs. N-HLF
TGFβ3	1.755
HAS-2	2.062
TWIST1	2.425
WNT2	3.052
SFRP2	3.695
RSPO2	4.331
TBX4	4.593
ITGA8	4.874
CHI3L1	5.027
HHIP	5.09

## Supplementary Materials

Supplementary materials can be accessed at https://www.mdpi.com/2073-4409/9/6/1371/s1. Figure S1: SHG analysis of N-HLF and CF-HLF sheets, Figure S2 2D molecular analysis (CF-HLF vs N-HLF), Figure S3 2D and 3D molecular analysis (CF-HLF vs N-HLF), Figure S4 Specific 2D molecular analysis, Figure S5 Young N-HLF produced an high quantity of collagen in microtissues; Supplementary table S1: Gene Ontology enrichment analysis of the differentially expressed genes (DEGs) into the 3D dataset (GSE141536), Supplementary table S2: Gene Ontology enrichment analysis of the differentially expressed genes (DEGs) into the 2D dataset (GSE141535), Supplementary table S3: list of DEGs resulting from the comparison of the 2D and the 3D datasets, Supplementary Table S4: GOEA and KEGG pathway analysis on the list of induced genes specific in 2D, 3D and in common; Supplementary table S5 Up-regulated genes from the 3D dataset (CF-HLF vs N-HLF) belonging to the GO Term “Mesenchymal cell differentiation” (GO:0048762) and EMT related.
