# Supplementary material for: Intrinsic Abnormalities of Cystic Fibrosis Airway Connective Tissue Revealed by an In Vitro 3D Stromal Model
Source: Cells. 2020 Jun 1;9(6):1371. doi: 10.3390/cells9061371 (PMC7348935; doi:10.3390/cells9061371)
Supplement: Supplementary file 1 [file cells-09-01371-s001.pdf]

Supplementary information

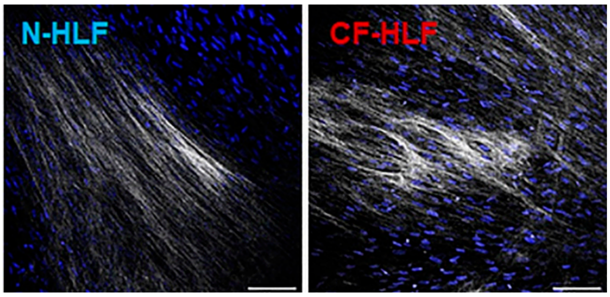

Figure S1 SHG analysis of N-HLF and CF-HLF sheets: In gray the endogenous collagen produced by fibroblasts and in blue the nuclei of the cells. Scale bar 100µm.

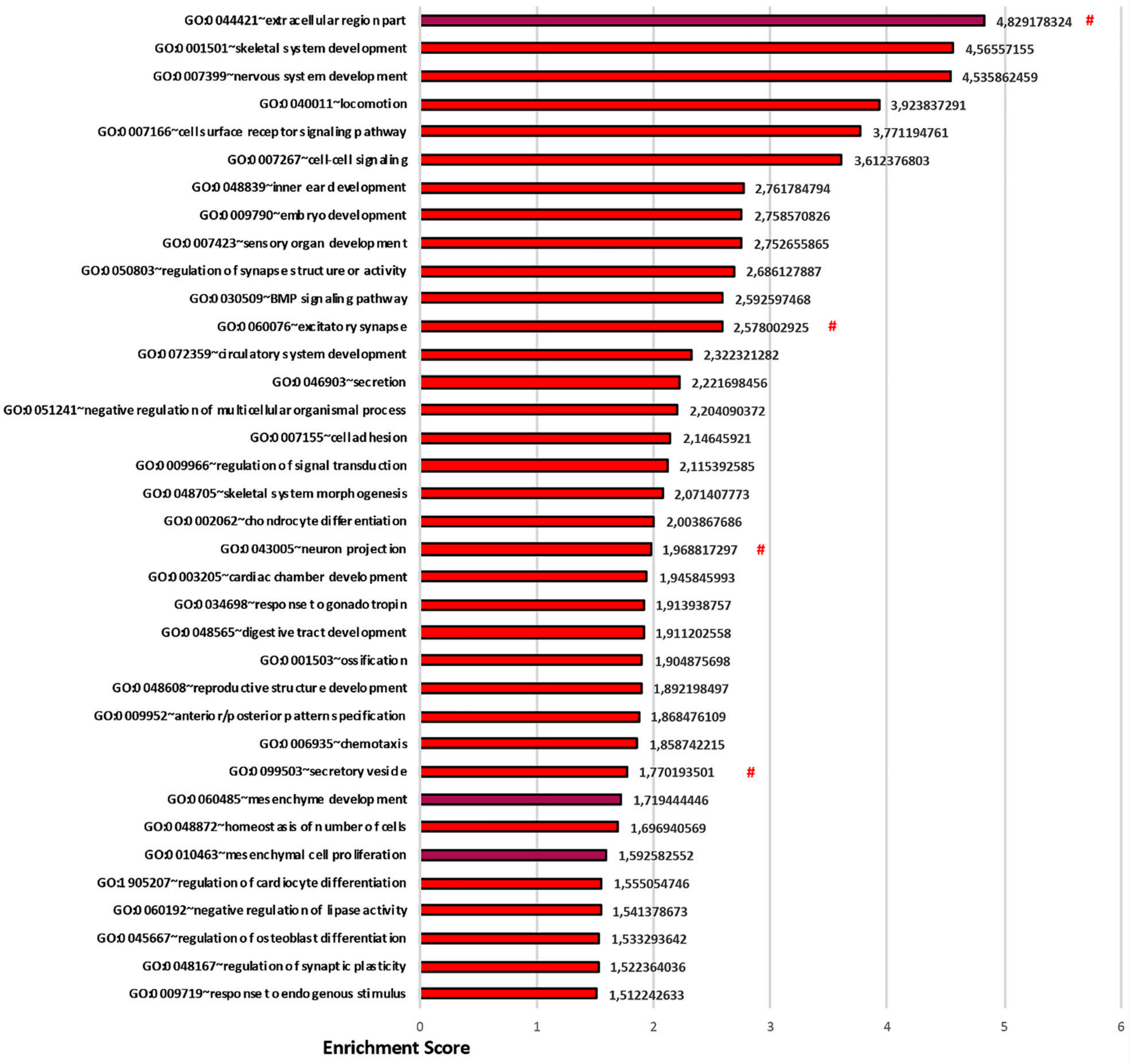

Figure S2 2D molecular analysis (CF-HLF vs N-HLF): BP and CC significant terms in which the DEG induced into the 2D dataset are mainly enriched. The threshold of induction plotted is logFC>2 (459 DEG, GSE141535). # are CC terms. Dark Red terms are the mainly interested induced terms found in our study.

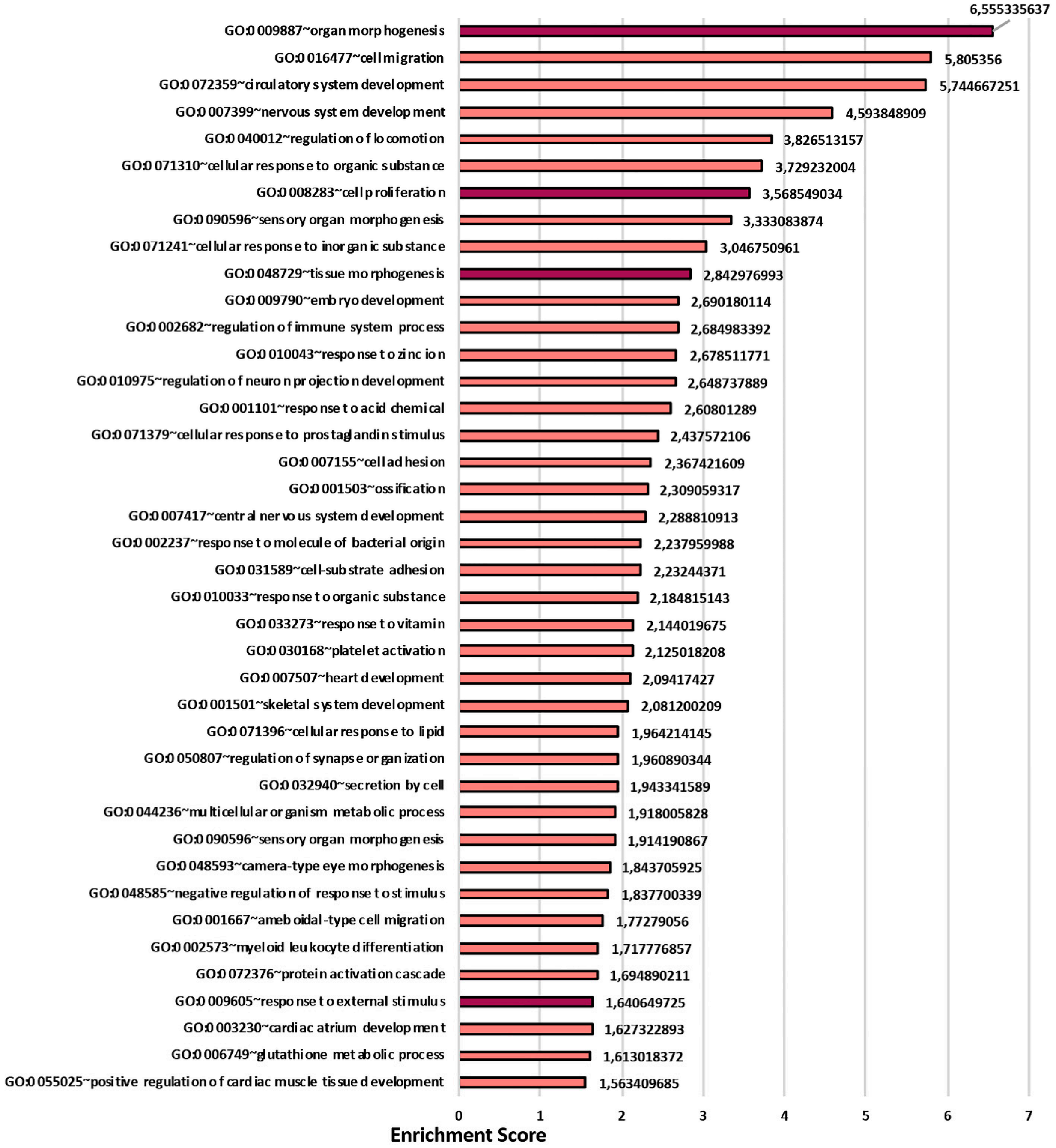

Figure S3 2D and 3D molecular analysis (CF-HLF vs N-HLF): All BP significant terms in which the DEG induced both in 2D and 3D datasets are mainly enriched. Dark Red terms are the mainly interested induced terms found in our study.

A)

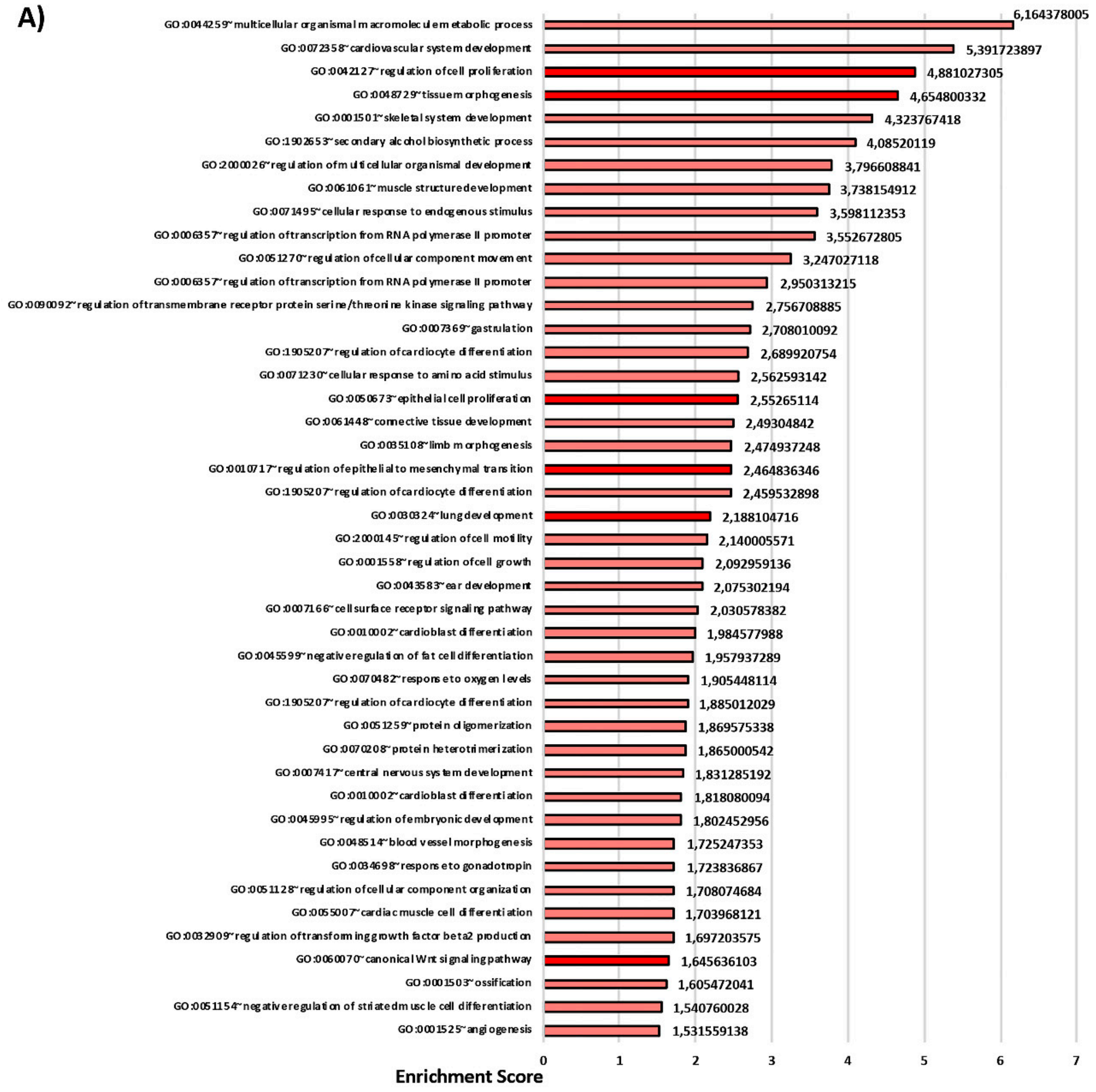

B)

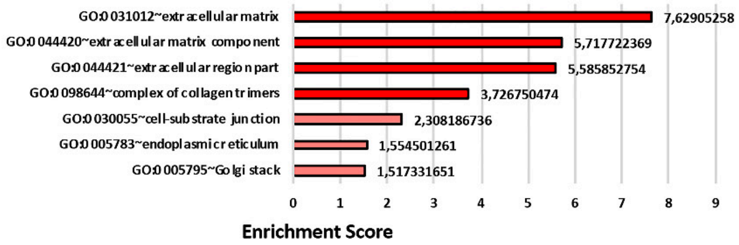

C)

Annotation Cluster 1	Enrichment Score: 4.6604658594421675	
Category	Term	Count
GOTERM_MF_FAT	GO:0046872~metal ion binding	203

Figure S4 Specific 2D molecular analysis: A) BP and B) CC significant terms in which the DEG specifically induced (779) into the 2D dataset are mainly enriched. Dark Red terms are the mainly interested induced terms found in our study C) The metal ion binding is the top MF in this dataset and is not enriched in the 3D specific induced dataset.

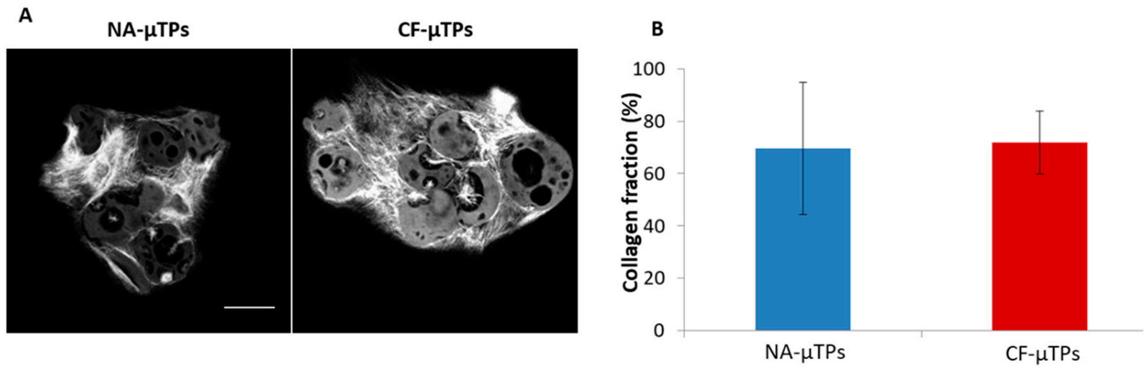

Figure S5 Young N-HLF produced an high quantity of collagen in microtissues: A) Second Harmonic Generation (SHG) imaging of collagen fibers produced by NHLF from a 3 years old child and DHLF from a 45 years old adult in normal and cystic fibrosis microtissues (NA-µTPs and CF- µTPs), Scale bar 100µm; B)Graphic of collagen fraction (%) in NA-µTPs and CF- µTPs, data are showed as mean ± standard deviation.

Table S5 Up-regulated genes from the 3D dataset (CF-HLF vs N-HLF) belonging to the GO Term "Mesenchymal cell differentiation" (GO:0048762) and EMT related

Gene symbol	logFC_CF-HLF *vs* N-HLF
FAM83D	5.817
WNT2	3.052
MSX2	2.011
SFRP2	3.695
HAS2	2.062
TMEM100	2.47
AXIN2	3.08
BMP7	4.212
TWIST1	2.425
